# Novel fast analytical method for indirect determination of MCPD fatty acid esters in edible oils and fats based on simultaneous extraction and derivatization

**DOI:** 10.1007/s00216-017-0381-z

**Published:** 2017-05-08

**Authors:** Renata Jędrkiewicz, Agnieszka Głowacz-Różyńska, Justyna Gromadzka, Piotr Konieczka, Jacek Namieśnik

**Affiliations:** Department of Analytical Chemistry, Faculty of Chemistry, Gdańsk University of Technology, 11/12 Narutowicza Street, 80-233 Gdańsk, Poland

**Keywords:** Foods/beverages, Organic compounds/trace organic compounds, GC, Quality assurance/control, Simultaneous extraction and derivatization, MCPD esters

## Abstract

A novel method for indirect determination of MCPD esters levels in lipid samples has been developed. The method is based on combination of extraction and derivatization in the same sample preparation step. It is achieved by the application of diethyl ether as extraction solvent for isolation of analytes released from esterified forms from the water phase and as dilution solvent for solid PBA – the derivatization agent. It is a noteworthy improvement of recommended indirect approaches available in the literature because such steps as sample clean-up, multiple liquid–liquid extractions, and preconcentration are excluded in the proposed solution. In this way, the developed procedure is shortened and simplified. Such an approach also minimizes the utilization of organic solvents; therefore, it is in accordance with the principles of “green analytical chemistry.” In spite of the fact that the step of sample clean-up is omitted, no deterioration in GC-MS system performance was observed. Equivalence testing of the developed procedure and AOCS cd 29b-13 official method (SGS) has been conducted. It was concluded that results obtained by both methods do not significantly differ statistically. The procedure has been applied to determination of MCPD esters concentrations in lipid fractions isolated by accelerated solvent extraction technique from such foodstuffs as bakery products, salty deep-fried snacks, and instant products. In all investigated samples, the level of bound MCPD was elevated. Additionally, for both procedures, the environmental impact (with the use of analytical Eco-scale) and uncertainty budget have been assessed and compared.

## Introduction

Chloropropanols are the group of chlorinated derivatives of glycerol known as food contaminants since 1978, when their presence was discovered in acid hydrolyzed vegetable protein (HVP) used as food additive for flavor enhancement [1]. Among these compounds, 3-monochloropropane-1,2-diol (3-MCPD) has been investigated intensively regarding its possible toxic effect on living organisms. Short-term and long-term toxicity studies carried out on rodents proved nephrotoxicity and testicular toxicity after taking in regular doses of 3-MCPD by rats [2–4].

Monochloropropanediols regained global attention in the past few decades in the food safety field with the discovery of elevated levels of 3-monochloropropane-1,2-diol (3-MCPD) and 2-monochloropropane-1,3-diol (2-MCPD) bound in the forms of fatty acid esters in different foods [5, 6]. By now, elevated levels of MCPD esters have been discovered in refined edible oils (the highest amounts in refined palm oil), foodstuffs containing refined oils in their composition (such as powdered infant formulas, margarines, and various types of palm oil-containing products), and deep-fried products [7–11]. This finding allowed for the assumption (proven later in the research) that the formation of MCPD esters is enhanced by the elevated temperature applied in the refining process of oils or industrial/domestic food processing (frying or smoking) [12–14]. The concern related to the presence of MCPD esters in different foods is bound to the possible release of free chlorinated propanols from their esterified form during digestion and their further metabolism to β-chlorolactaldehyde and β-chlorolactic acid, which may cause nephrotoxicity and antifertility [15–17].

Monochloropropanediols, especially 3-MCPD, are in the field of interests of European associations dealing with food safety for over a decade [18]. In 2001, the European Commission’s Scientific Committee on Food (SCF) classified 3-MCPD as a non-genotoxic, threshold carcinogen. In 2004, the Joint FAO/WHO Expert Committee on Food Additives (JECFA) established the tolerable daily intake (TDI) for 3-MCPD at a level of 2.0 μg per kg of body weight per day after taking into consideration the lowest observed effect level (LOEL) in toxicity studies on rodents [19]. German Federal Institute for Risk Assessment published in 2007 and 2013 important data regarding the bound (esterified) form of MCPD [20, 21]. After identifying the problem of MCPD esters presence in food and hindered risk assessment regarding this phenomenon, European Authorities obliged scientific and industrial representatives to collaborate with the general aim of minimizing the possibility of MCPD esters formation during food production and processing [18]. In addition, European Food Safety Authority (EFSA) released in 2016 an opinion on the risk on human health related to the presence of MCPD esters in various food products investigated with regard to different age groups, and the CONTAM panel lowered the TDI value for 3-MCPD to 0.8 μg per kg of body weight per day [22]. The same opinion contains recommendation for further research on consumer exposure to MCPD esters through food.

In order to monitor 3-MCPD and 2-MCPD esters levels in edible oils and fats, several analytical approaches have been developed so far. Among them, two general analytical approaches for MCPD esters determination may be distinguished: direct and indirect [23]. Direct approach is based on the identification and quantification of all different ester species (monoesters and diesters, homoesters and heteroesters) which is useful for assessing the possible human health risk related to biological fate and toxic activity of specific esters species [24]. This approach is also quite simple when it comes to sample preparation procedure because before LC or SFC analysis only solid phase extraction or basic dilution is needed, without any time-consuming conversion of the analytes [25]. On the other hand, direct methodology is considered as rather expensive and challenging because of the need of numerous analytical standards (over 30 different compounds) and complicated chromatographic separation (similar structures of compounds to be separated including positional isomers) [26]. For these reasons, indirect procedures are preferred by analytical chemists to be utilized for routine analysis. Indirect approach is based on the transesterification reaction in which MCPD esters are released into the form of free monochloropropanediols, followed by sample clean-up, derivatization reaction, and GC analysis [27, 28]. This approach suffers mainly from a quite extensive sample preparation process with 16-h transesterification reaction and several liquid–liquid extractions applied for sample clean-up [29]. The release of free MCPD from esterified form needs to be carried out under smooth conditions and therefore takes several hours because this prevents he conversion of diols into oxiranes and, as a result, underestimation of the MCPD esters content in the sample. In this situation, to modify the indirect approach in such a way as to develop a methodology that is faster and easier to be implemented in routine analytics, sample preparation steps after the hydrolysis of MCPD esters present originally in the lipid sample (oil/fat or lipid fraction isolated from food product) were investigated.

This paper presents a novel indirect procedure for fast monitoring of MCPD esters in lipid samples such as edible oils, fish oils, or lipid fractions isolated from a wide variety of food products. The general advantage of developed procedure in comparison to indirect methodologies available in the literature is that after hydrolysis of the esters, further preparation of the sample prior to GC-MS analysis has been shortened and simplified. Instead of multiple liquid–liquid extractions for both sample clean-up and extraction of released MCPDs (present at this stage in the water phase) and derivatization of the analytes, these processes were combined into one step. It was achieved by using diethyl ether both as extracting solvent and solvent for diluting the derivatization agent – solid phenylboronic acid (PBA). The described procedure has been successfully applied for the determination of 3-MCPD and 2-MCPD esters in lipid fractions isolated from such food products available in the market as bakery products, deep-fried products, and instant products in order to assess the exposure of the consumers to investigated compounds. The results were compared with those obtained with the use of so-called “SGS 3-in-1” method in the literature [27], one of the official AOCS methods – AOCS cd 29b-13 [30] recommended for monitoring MCPD esters in lipids. Both methods have been compared according to validation parameters, results obtained from analysis of real samples, environmental impact, and uncertainty budget. It should be emphasized that this paper reports for the first time the evaluation of indirect MCPD esters measurement with regard to the issues related to uncertainty of analytical methodology and its environmental impact. The developed indirect methodology has been proven to be a very useful tool for monitoring of MCPD esters in lipid food samples. Introduced modifications contributed to the development of a procedure perfectly suitable for rapid routine analysis, where multiple operations with the sample are not desired. The procedure merges the advantages of both indirect and direct methodologies currently available in the literature – it is still easier in case of chromatographic separation (different ester species are converted into two compounds that are determined) but at the same time it is also characterized by fast and simple sample preparation before GC analysis. It should be emphasized that the improvements in MCPD esters analytics is desired taking into account EFSA recommendation from the last year on the need of constant monitoring of MCPD esters levels in food products available for the consumer.

## Materials and methods

### Chemicals and samples

1,2-Bis-palmitoyl-3-chloropropanodiol, 1,2-bis-palmitoyl-3-chloropropanediol-d_5_, 1,3-distearoyl-2-chloropropanediol and 1,3-distearoyl-2-chloropropanediol-d_5_ were purchased from Toronto Research Chemicals (Toronto, Canada). Diethyl ether, methanol, dichloromethane, hexane, sodium hydroxide, sodium bromide, anhydrous sodium sulphate, orthophosphoric acid (85%), ethyl acetate,, phenylboronic acid (PBA), and acetone were purchased from Sigma Aldrich (St. Louis, MO, USA). Compressed gases as nitrogen and helium were purchased from Linde Gas (Munich, Germany). All chemicals used were of analytical grade.

The solutions used in the sample preparation procedure were prepared in the following way:

1. Transesterification solution: sodium hydroxide in methanol solution was prepared by dissolving 250 mg of NaOH in 100 mL MeOH.
2. Neutralizing solution: 50 g of sodium bromide was dissolved in 100 mL of deionized water, the solution was slightly acidified by addition of 3.5 mL of orthophosphoric acid.
3. Extraction and derivatization solution: 17 g of PBA was dissolved in 10 mL of diethyl ether.
4. Derivatization solution (for “SGS 3-in-1” method): 200 mg of phenylboronic was dissolved in 10 mL of diethyl ether.
5. Stock solutions of deuterated and non-deuterated standards: solid, pure 1,2-bis-palmitoyl-3-chloropropanodiol and 1,3-distearoyl-2-chloropropanediol were dissolved in ethyl acetate to the concentration of 5 mg mL^–1^ each; solid, pure 1,2-bis-palmitoyl-3-chloropropanediol-d_5_ and 1,3-distearoyl-2-chloropropanediol-d_5_ were dissolved in ethyl acetate to the concentration 5 mg mL^–1^ and 2 mg mL^–1^ each.
6. Spiking solutions of deuterated and non-deuterated standards: stock solutions were diluted with ethyl acetate to the desired concentrations – 5 μg mL^–1^ and 50 μg mL^–1^ of non-deuterated standards and 50 μg mL^–1^ of deuterated standards.

Investigated food products (18 samples) are listed below:

- cookies (five samples)
- salty deep-fried snacks such as potato chips, crisps, crackers (five samples)
- instant sauces, soups, and noodle soups (eight samples)

All products were purchased in the local market. The content of lipid fraction in investigated samples according to the label given by the manufacturer (and confirmed in the research) was in the range of 15–35%. It consisted mainly of refined palm oil and rapeseed oil.

### Sample preparation procedure

All solid food products were crushed and homogenized. In case of instant products with noodles, crushed noodles were mixed with flavoring powder attached to the product.

Ten g of crushed product were mixed thoroughly with diatomaceous earth and placed in a stainless steel cell. Lipid fraction isolation was carried out with the use of accelerated solvent extraction (ASE) by dichloromethane/methanol solution (77% dichloromethane in methanol). The organic solvent was evaporated from the extract with the use of a rotary evaporator to obtain pure lipid fraction. The extraction procedure has already been developed and validated on powdered infant formula samples in previous studies [11].

The smooth alkaline transesterification procedure applied in this work was developed and published in 2011 [27]. Carrying the reaction in “SGS way” (slightly alkaline solution and lowered temperature) allows for complete release of free MCPD from its esterified form with minimized possibility of MCPD transformation into oxiranes (especially glycidol), which is a favored reaction in strong alkaline conditions and room- or elevated temperature.

**Fig. 1:**
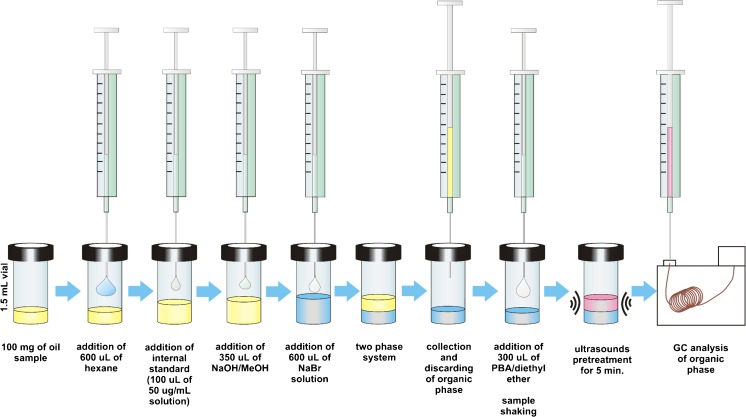
Schematic representation of developed procedure based on simultaneous extraction and derivatization

The sample preparation procedure of isolated lipid fraction is presented in Fig. 1.

In brief, 0.1 g of lipid sample was melted and homogenized if necessary and dissolved in hexane. After mixing the sample to obtain homogenous solution, internal standard spiking solutions were added. Both sample and transesterification solutions were cooled down to –25 °C. Then the transesterification reaction was carried out by adding the NaOH in MeOH solution to the sample, and the sample was incubated in –25 °C for 16 h. The reaction was stopped by the addition of neutralization solution. The upper organic phase was discarded. The water phase containing released MCPDs was extracted by the solution of PBA in diethyl ether, carrying out the derivatization reaction at the same time. Both sample and PBA solution were cooled down in a freezer before this step to avoid the evaporation of diethyl ether and changes in the concentration of the extract. The mixture was shaken vigorously for 1 min and left in ultrasounds for 5 min at room temperature. Subsequently, the sample was cooled down in a freezer once again for the same reason as described above and then the upper extract was transferred into the GC vial with 300 μL insertion and analyzed utilizing the GC-MS technique.

For comparison, the same samples were prepared in accordance with “SGS 3-in-1” methodology. After transesterification reaction and mixture neutralization (carried out in the same way as mentioned above) the organic phase was concentrated under a gentle stream of nitrogen and after that the sample was extracted two times by 600 μL of hexane (the organic phase was discarded). Aqueous phase was extracted three times by 600 μL of diethyl ether and ethyl acetate mixture (3:2). Collected extracts were dried on anhydrous sodium sulphate; 80 μL of PBA solution was added to extracts and derivatization reaction was carried out in ultrasounds for 5 min. Then the sample was evaporated to dryness under a gentle stream of nitrogen, and the dry residue derivatives were redissolved in 300 μL of isooctane and quantified using GC-MS.

### Instrumentation and quantification principle

Both simultaneous extraction and derivatization procedure and “SGS 3-in-1” procedure were carried out with the use of the same instrumentation. Accelerated solvent extraction was carried out with the use of ASE 350 equipment (Dionex, Sunnyvale, CA, USA). Applied extraction conditions were as follows: 120 °C, 1500 psi, 5 min of static time, one extraction cycle.

GC-MS analysis was carried out by Agilent Technologies Gas Chromatograph 7890A coupled with Agilent Technologies Mass Spectrometer 5977C with the separation of analytes on Agilent Technologies chromatographic column DB5-MS (30 m; id: 0.25 mm; film thickness: 0.25 μm, stationary phase: 95% PDMS, 5% phenyl groups) (Agilent Technologies, Santa Clara, CA, USA). Pre-column of 2 m fused silica was installed. As a carrier gas, helium 6.0 was used with constant flow equal to 1.2 mL min^–1^. One μL of extract was injected in splitless mode. GC oven temperature program was as follows: 40 °C with an increase of 6 °C min^−1^ to 190 °C, followed by increase of 30 °C min^−1^ to 260 °C held for 10 min. Transfer line temperature was equal to 280 °C, ion source and quadrupole temperature in mass spectrometer were equal to 250 °C and 150 °C, respectively.

Qualitative and quantitative analysis by mass spectrometer was carried out by monitoring target and qualifier ions in SIM mode. The following ions were monitored: for 3-MCPD-derivative ions at *m/z* 147 (target), *m/z* 196 and 198 (qualifiers); for 3-MCPD-d_5_-derivative ions at *m/z* 150 (target), *m/z* 201 and 203 (qualifiers); for 2-MCPD-derivative ions at *m/z* 196 (target) and *m/z* 198 (qualifier); for 2-MCPD-d_5_-derivative ions at *m/z* 201 (target) and *m/z* 203 (qualifier). Quantification principle was based on the signal area ratios of 3-MCPD/d_5_-3-MCPD and 2-MCPD/d_5_-2-MCPD derivatives.

## Results and discussion

### Simultaneous extraction and derivatization – principle and method development

**Fig. 2:**
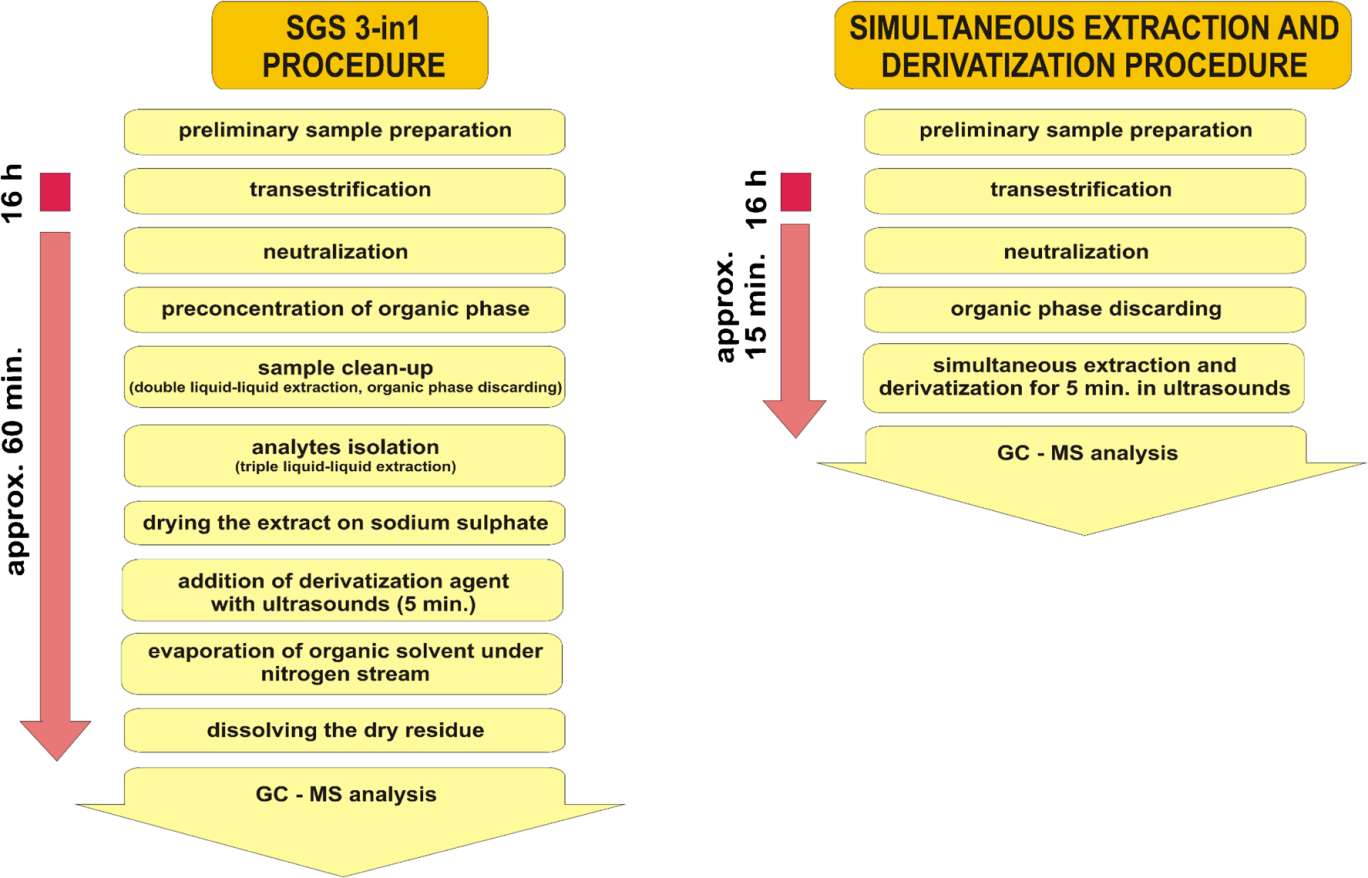
Comparison of “SGS 3-in-1” indirect procedure and developed procedure based on simultaneous extraction and derivatization

In order to simplify indirect procedures recommended by AOCS [30, 31], which involve multi-step sample preparation process consisting of multiple-extractions, after transesterification and neutralization reaction, simultaneous extraction and derivatization step was applied. The comparison of “SGS 3-in-1” indirect procedure and developed approach is presented in Fig. 2.

The proposed procedure is aimed at avoiding such individual sample preparation steps as:

- liquid–liquid extraction for sample clean-up
- liquid–liquid extraction for analytes isolation from water phase
- derivatization
- preconcentration of the analytes in the final extract by organic solvent evaporation

The solution with combining extraction and derivatization in the same step not only shortens and simplifies the entire procedure, but also reduces dramatically the use of organic solvents. Therefore, the procedure is in accordance with “green analytical chemistry” principles. Such approach also contributes significantly to lowering the costs of single analytical cycle. Even though sample clean-up step was excluded, no deterioration of GC-MS system performance was observed. It is related to the fact that most of the lipid components that could contaminate the system are easily soluble in nonpolar solvents so they are removed with the hexane phase discarding after neutralization. The amount and concentration of PBA solution in diethyl ether was selected, taking into consideration the amount of PBA needed to be in excessive ratio to the analytes (to enhance the formation of the derivative) and the volume of GC vial insert (300 μL). It allows for transferring the extract directly to the GC analysis without the preconcentrating/dissolving step. The influence of time of carrying out the simultaneous extraction and derivatization process was investigated. The samples were placed in ultrasounds to enhance the process for 5 min, 15 min, 30 min, and 60 min. After 5 min of simultaneous extraction and derivatization, the process was complete (recoveries close to 100%, see Table 1) so further research was carried out in this way. The conditions of alkaline transesterification and neutralization step have not been optimized, as it has already been done and investigated in detail [27].

**Table 1:** The summary of data on validation parameters of applied procedures

		Standards	Linearity	LOD [mg kg^–1^]	LOQ [mg kg^–1^]	Range [mg kg^–1^]	Precision	Trueness Rec ± u (k = 2) [%]	Expanded uncertainty U (k = 2) [%]
Bound 3-MCPD	SGS method	0.1–1.0 Concentrations: 0.1; 0.3; 1.0	y = 0.0937x + 0.0073 R = 0.995	0.034	0.10	0.10–10	CV standards 2.8% CV sample 1.8%	96.7 ± 5.0	9.9
		1.0–10 Concentrations: 1.0; .3.0; 5.0; 10	y = 0.1156x – 0.0547 R = 0.997						
	Simultaneous extraction & derivatization method	0.1–1.0 Concentrations: 0.1; 0.3; 1.0	y = 0.1074x + 0.0043 R = 0.998	0.014	0.043	0.043–10	CV standards 3.5% CV sample 2.5%	100.1±5.0	9.1
		1.0–10 Concentrations: 1.0; .3.0; 5.0; 10	y = 0.1100x – 0.0008 R = 0.999						
Bound 2-MCPD	SGS method	0.10–1.0 Concentrations: 0.1; 0.3; 1.0	y = 0.0753x + 0.0021 R^2^ = 0.998	0.0084	0.025	0.025–10	CV standards 1.6% CV sample 1.6%	100.1 ± 5.0	6.3
		1.0–10 Concentrations: 1.0; .3.0; 5.0; 10	y = 0.0826x + 0.0021 R^2^ = 0.999						
	Simultaneous extraction & derivatization method	0.10–1.0 Concentrations: 0.1; 0.3; 1.0	y = 0.0719x + 0.0031 R = 0.995	0.017	0.052	0.052–10	CV standards 4.0% CV sample 2.2%	99.8 ± 5.2	10
		1.0–10 Concentrations: 1.0; .3.0; 5.0; 10	y = 0.0841x – 0.0104 R = 0.999						

### Determination of validation parameters in MCPD esters measurement

It is believed that foods other than soy sauces and related products (containing only free MCPDs) and edible oils and fats (containing only esterified MCPDs) may contain both free and bound MCPDs, but bound MCPDs are present mainly in lipid fractions of these foods. This may be assumed on condition that MCPD esters take the form of lipid-like compounds, mimicking in their structure natural acylglycerols and, therefore, by the complete isolation of lipid fraction, MCPD esters are believed to also be isolated effectively. The lipid fraction isolation procedure has already been optimized and validated in previous studies [11] in powdered infant formula samples. Optimized conditions allowed for the isolation of more than 95% of lipid fraction contained in the solid food sample. Recovery test on spiked blank powdered infant formula sample proved the effective recovery of MCPD esters (recoveries 97–107%) with complete isolation of the lipid fraction. In the research of the application of simultaneous extraction and derivatization procedure to the determination of MCPD esters in lipid fractions isolated from various solid foodstuffs, the same ASE procedure was utilized. However, it should be mentioned that in this study, investigated food products are of different matrix composition than powdered infant formulas studied previously. For this reason it should be stated that some minor amounts of MCPD esters could be incorporated in complex food matrix in such a way that they were not completely isolated with the total isolation of lipid fraction. The discussion presented in the following section corresponds to the methodology of MCPD esters determination in lipid samples (edible oils, fats, or lipid fractions isolated from other foods).

Calibration was carried out with the application of internal standard methodology. Validation data were achieved by analyzing spiked samples. Spiking with the standards was applied directly to the lipid matrix. In this research, for blank sample, cold-pressed and non-refined evening primrose oil was selected. According to previous studies [10], the level of MCPD esters in such oil is not detectable.

The validation procedure was the same for proposed novel solution (with simultaneous extraction and derivatization) and for “SGS 3-in-1” method applied as a comparative method. LOD was determined from the calibration curve according to the formula:

$$ L O D=3.3 S/ b, $$

where S is the arithmetical mean of SD of intercept and residual SD; and b is the slope

The LOQ value was counted as tripled LOD value. The performance of LOQ was confirmed by the analysis of sample spiked with this concentration. The signal to noise ratio in this case was higher than 10. The spiking levels of 1,2-bis-palmitoyl-3-MCPD and 1,3-distearoyl-2-MCPD for calibration curves were in the range of 0.53–52.5 and 0.57–57.5 mg kg^−1^ correspondingly (equal in both cases to 0.10–10 mg kg^−1^ released MCPD). The analysis of spiked samples for calibration was carried out in triplicate. The curves were plotted as a peak area ratio of MCPD/d_5_-MCPD derivatives in the function of spiking level of bound MCPD. Precision (expressed as repeatability) was estimated with regard to analysis of spiked samples for calibration (six concentration levels: 0.1, 0.3, 1.0, 3.0, 5.0, and 10.0 mg kg^−1^ released MCPD, n = 3 for each concentration) and blank oil sample spiked with the level of released MCPD equal to 5.0 mg kg^−1^ (n = 5). Trueness (expressed as recovery) was determined for blank sample spiked with the concentration of released MCPD equal to 5.0 mg kg^−1^ (n = 5). Expanded uncertainty (1) reflects all the sources of uncertainty, which have an impact on the final result of MCPD esters analysis. These are (all as relative values): (2) standard uncertainty related to precision (expressed as repeatability), (3) uncertainty related to calibration procedure, (4) trueness, and (5) LOD determination, in accordance with the following equations:

1 $$ {U}_x= k\sqrt{u_{prec}^2+{u}_{cal}^2+{u}_{true}^2+{u}_{LOD}^2} $$

2 $$ {u}_{prec}=\sqrt{\frac{{\displaystyle {\sum}_{i=1}^n C{V_i}^2}}{n}} $$

3 $$ {u}_{cal}=\frac{S{ D}_{x, y}}{b}\cdot \sqrt{\frac{1}{p}+\frac{1}{n}+\frac{{\left({x}_{p r}-\overline{x}\right)}^2}{Q_{x x}};}{Q}_{x x}={\displaystyle {\sum}_{i=1}^n{\left({x}_i-\overline{x}\right)}^2} $$

4 $$ {u}_{true}=\sqrt{{\left(\frac{C{ V}_{rec}}{\sqrt{n}}\right)}^2+{u}_{std}^2} $$

5 $$ {u}_{LOD}=\frac{LOD}{x} $$

where CV is the coefficient of variance; n is the number of measurements; SD_x,y_ is the standard deviation of calibration curve intercept; b is the calibration curve slope; p is the total number of standard solutions used for calibration curve preparation; *x* is the result; $\overline{x}$ is the average concentration of the standard solution used for calibration curve preparation; u_std_ is the uncertainty of standard solution preparation; rec is the recovery.

The summary of obtained validation data is summarized in Table 1.

### Uncertainty budget and greenness evaluation by analytical Eco-scale

**Fig. 3:**
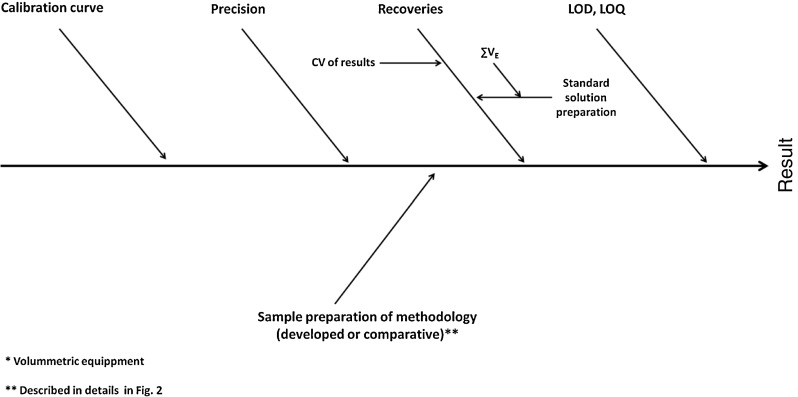
Ishikawa diagram representing the parameters that influence the final result of MCPD esters analysis

The parameters that influence the result of MCPD esters analysis by indirect methodology are presented via Ishikawa diagram in Fig. 3. Uncertainty budget of analytical method presents the uncertainty contribution of individual metrological parameters described in Eqs. 2–5 to the expanded uncertainty of the method described in Eq. 1). Appropriate equations have been presented in previous section.

**Fig. 4:**
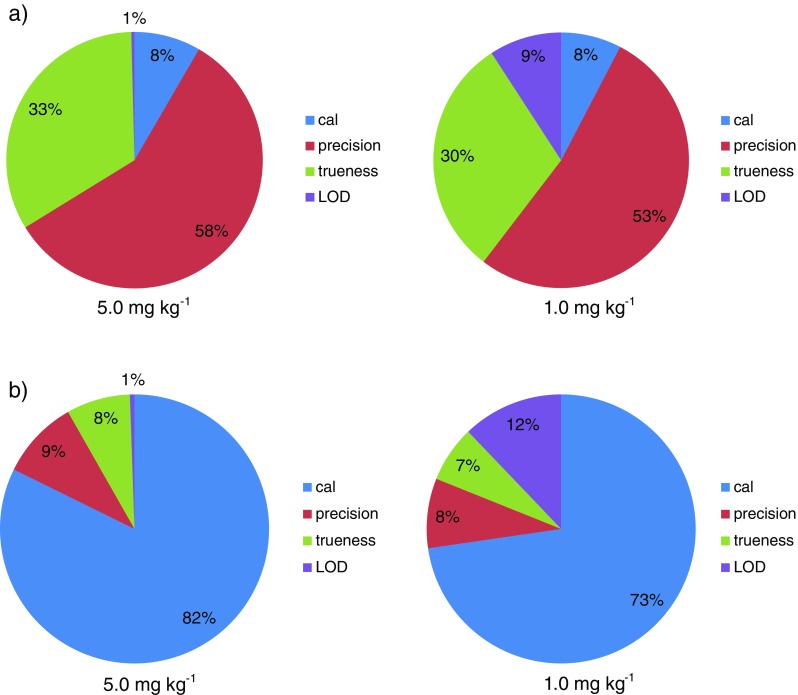
Uncertainty budget estimated for: (**a**) bound 32-MCPD determined by developed method; (**b**) bound 3-MCPD determined by control method

Figure 4 presents the comparison of uncertainty budgets estimated for bound 3-MCPD determined with the application of both methods. Based on the presented graphs, it may be concluded that the individual uncertainty contributions of selected metrological parameters differ between each other comparing developed and control method. The main difference is related to the contribution of calibration and precision uncertainty. The results obtained by the developed methodology seem to be slightly affected by calibration uncertainty but highly affected by precision uncertainty. In the case of control method, the trend is the opposite. The reason why precision plays an important role in the case of the developed simultaneous extraction and derivatization procedure may be related to the fact that transferring the diethyl ether phase between the vials is difficult to be carried out in a repeatable way – diethyl ether is a highly volatile solvent with low surface tension. The insignificant contribution of LOD in the case of both methods estimated for sample containing bound 3-MCPD at a level of 5.0 mg kg^−1^ is not surprising because this value highly exceeds determined LOD value. Uncertainty budget estimation for 1.0 mg kg^−1^ concentration revealed that the lower the analyte concentration (closer to LOD value), the higher the LOD uncertainty contribution to the expanded uncertainty of the methodology.

The analytical Eco-scale [32] is based on assigning penalty points to parameters of an analytical process that are not in agreement with the ideal green analysis. This comprehensive semiquantitative tool allows for the assessment of the greenness of new or modified methods. It has been applied for the greenness evaluation of the developed within this research simultaneous extraction and derivatization method and the control, SGS method (Table 2).

**Table 2:** The penalty points (PPs) for bound MCPD determination in food lipids by developed and control methodologies

	Penalty points	
	SGS (control method)	Simultaneous extraction & derivatization (developed method)
Reagents		
Diethyl ether	4	4
Isotopically labeled IS	6	6
Methanol	6	6
Sodium hydroxide	2	2
Hexane	8	8
Ethyl acetate	4	4
Phenylboronic acid	1	1
Isooctane	8	0
Instruments		
GC-MS technique	2	2
Occupational hazard	3	0
Waste	1	1
Total penalty points	45	34
Analytical Eco-scale total score	55	66

Eco-scale score estimated for both methodologies (100-x where x is the sum of total penalty points) and compared with the ranking [32] represents acceptable green analysis in both cases. The significant difference between the methods with regard to analytical Eco-scale is the application of isooctane for dilution of the dry residue of the final extract before GC analysis in SGS method. Another difference is the occupational hazard assessed regarding the emission of vapors to the air, which takes place in the SGS method while evaporating the final extract to dryness. This, however, does not have a significant impact on greenness evaluation of both methods with the application of analytical Eco-scale.

### MCPD fatty acid esters determination in lipid fractions isolated from investigated solid food products – method application

**Fig. 5:**
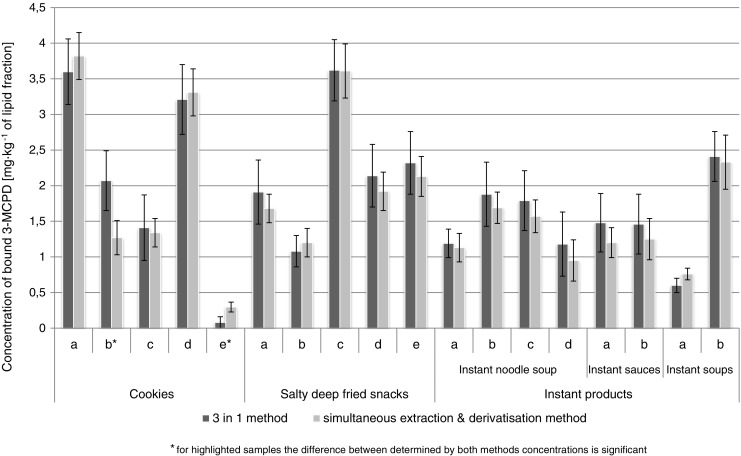
The levels of bound 3-MCPD in lipid fractions isolated from investigated food products determined by simultaneous extraction and derivatization procedure and “SGS 3-in-1” procedure

**Fig. 6:**
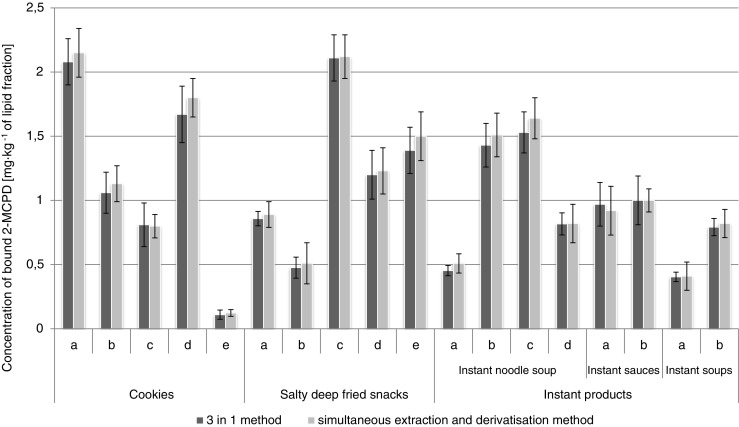
The levels of bound 2-MCPD in lipid fractions isolated from investigated food products determined by simultaneous extraction and derivatization procedure and “SGS 3-in-1” procedure

In order to verify the applicability of proposed solution to the monitoring of MCPD esters levels in lipid fractions isolated from foodstuffs available to consumers, selected products purchased in the local market were investigated. Levels of MCPD esters in isolated from investigated foods lipid fractions determined by the developed procedure based on simultaneous extraction and derivatization and the comparative “SGS 3-in-1” method are presented in Figs. 5 and 6. Error bars presented in the graphs were calculated as expanded uncertainty, taking into consideration not only the standard deviation (repeatability) of concentration measurements carried out in triplicate but also individual uncertainties (discussed above) affecting these measurements, in accordance with Eq. 1 mentioned above. In this way, the results obtained by both methods may be better compared and significant differences may be indicated (see asterisks in the graph presented in Fig. 5).

In all investigated samples, the level of MCPD esters was elevated, which confirms available literature data that the presence of refined oils (especially palm oil) is responsible for the highest levels of these compounds detected in different foodstuffs. In this study, the highest level (over 3.5 mg kg^–1^ isolated fat) was determined in bakery products. Interesting results may be observed for the group of instant products. To now, only free MCPD has been taken into consideration in case of soy sauces, dressings, seasonings, and dry preparations for soups and dishes [22]. Indeed, HVP and related products are likely to contain significant amounts of MCPD only in the free form. However, it should be noted that in the case of instant products such as for example noodle soups, the seasonings and dry preparations based on HVP are consumed together with the noodles, which contain refined palm oil. In this situation, the possible contamination of the product by esterified MCPD should not be underestimated. Obtained results indicate that in lipid fractions of instant products containing refined edible oils, the presence of bound MCPD may be significant – up to 2.4 mg kg^–1^ isolated fat. The concentration of bound 2-MCPD was lower than bound 3-MCPD in investigated samples.

### Method equivalence testing

Equivalence testing has been done first on the basis of validation parameters determined for both methods. According to the results of these parameters (Table 3) it is possible to conclude that the difference between methods is not statistically significant, taking into consideration trueness, precision (expressed as repeatability), LOD, and finally uncertainty. Variance investigation regarding the results of bound MCPD determination in real samples by both methods has been carried out by F-Snedecor test. Based on the results it is possible to conclude that the differences between investigated parameters are not statistically significant (calculated values of F parameters are 1.95 and 2.00, whereas critical value of F parameter is equal to 6.39).

**Table 3:** Data obtained within equivalence testing of developed and control methodologies

	Slope b	SD_b_	t_b_	Intercept a	SD_a_	t_a_	t_crit_
Bound 3-MCPD	0.992	0.063	0.127	–0.09	0.13	0.692	2.120
Bound 2-MCPD	1.040	0.019	2.105	–0.000	0.021	0.000	

Additionally, the regression method has been applied for comparison of the results obtained within the analysis of real samples with the application of the developed and control methods. The results obtained by the control method have been selected as reference values.

Based on statistical evaluation of the data of regression analysis (Student *t*-test used for it), it is possible to conclude that results obtained by both methods do not statistically differ significantly.

## Conclusions

A novel method for indirect determination of MCPD esters levels in lipid samples has been developed, validated, and applied for real samples. The method is based on a combination of extraction and derivatization in the same sample preparation step. It is achieved by the application of diethyl ether as extraction solvent for analytes isolation from water phase and dilution solvent for solid PBA – derivatization agent. The process of simultaneous extraction and derivatization was complete after 5 min treatment in ultrasounds. In comparison to recommended indirect approaches available in the literature, such steps as sample clean-up, multiple liquid–liquid extractions, and preconcentration are excluded in the proposed solution. In this way, the procedure developed by us is shortened and simplified; thus, it may be applied when rapid monitoring of MCPD esters level is needed (for example at each step of refining process of edible oils, which contributes to the formation of these compounds). Such approach also minimizes the utilization of organic solvents; therefore, it is in accordance with the principles of “green analytical chemistry.” Even though the step of sample clean-up was omitted, no deterioration in GC-MS system performance was observed. The developed procedure is characterized by satisfactory values of basic metrological parameters.

The comparison of the developed method and the control (SGS) was carried out. The methodologies were compared with regard to validation parameters, uncertainty budget, and environmental impact (by analytical Eco-scale method). Additionally, the statistical evaluation of both methods is similar with regard to validation parameters and bound MCPD content obtained within the analysis of real samples. On the basis of the results of the above mentioned evaluation, it is possible to conclude that both methods have satisfactory values of basic metrological parameters, represent acceptable green analysis, and do not significantly differ statistically.

The developed and control methodologies have been applied for determination of MCPD esters concentration in lipid fractions isolated by accelerated solvent extraction from such foodstuffs as bakery products, salty deep-fried snacks, and instant products. In all investigated samples, the level of bound MCPD was elevated, with the highest concentration determined in cookies (over 1000 μg kg^–1^ product). The presence of significant levels of bound MCPD in investigated foods is related to the fact the their lipid fractions consist mainly of refined palm oil and rapeseed oil. It is widely known that refined palm oil is highly contaminated by these toxicants. Obtained results indicate that MCPD esters may contaminate a wide range of food products, which may result in exceeding the TDI value, especially by humans of low body weight. Hence, the constant monitoring of these compounds is definitely needed. The developed simultaneous extraction and derivatization method is a tool designed for this purpose.
